# The causal role of multiple psycho-emotional disorders in gastroesophageal reflux disease: A two-sample Mendelian randomized study

**DOI:** 10.1371/journal.pone.0302469

**Published:** 2024-05-06

**Authors:** Jing Wang, Meng Song, Mingbo Cao

**Affiliations:** 1 Department of Gastroenterology, People’s Hospital of Zhengzhou University, Henan Zhengzhou, China; 2 Cancer Center, People’s Hospital of Zhengzhou University, Henan Zhengzhou, China; Institute of Medicine (IOM), Maharajgunj Medical Campus,TU, NEPAL

## Abstract

**Background:**

Observational studies have previously shown a potential link between psycho-emotional disorders, such as mood swings, highly strung, anxious feelings, and gastroesophageal reflux disease (GERD). However, the credibility of these associations could be influenced by various confounding factors. Consequently, our study sought to employ a Mendelian randomization (MR) approach to elucidate a potential causal relationship between psycho-emotional disorders and GERD.

**Method:**

Information on independent genetic variants linked to mood swings, highly strung, and anxious feelings was gathered from European populations participating in the IEU Open GWAS research. The FinnGen Consortium provided the genome-wide association study (GWAS) summary statistics for GERD. Our analysis employed the inverse variance weighted (IVW) method under the random effects model as the main analytical method. To further bolster our findings, we employed the weighted median and MR Egger methods. In addition, we conducted a series of sensitivity analyses.

**Results:**

Our study supports the existence of a causal relationship between psycho-emotional disorders and GERD. Mood swings, highly strung, and anxious feelings adversely affected GERD risk (mood swings: OR 2.21, 95% CI 1.19–5.59, p = 3.09 × 10–2; highly strung: OR 5.63, 95% CI 1.77–17.94, p = 3.42 × 10–3; anxious feelings: OR 2.48, 95% CI 1.08–4.33, p = 2.89 × 10–2).

**Conclusion:**

This Mendelian randomization study provides robust support for the notion that mood swings, highly strung and anxious feelings, are associated with an increased risk of developing GERD.

## Introduction

Gastroesophageal reflux disease (GERD) is one of the most common gastrointestinal disorders worldwide [1]. In the year 2005, about 20% of the population living in Europe and the US was estimated to be dealing with GERD [2]. Furthermore, it is imperative to acknowledge that the prevalence of this disease is presently on the ascent within developing nations [1]. Moreover, the protracted course of this ailment can precipitate a pronounced diminishment in patients’ quality of life, which will exert considerable economic strain on a global scale [3]. Numerous investigations have examined the intricate interplay between gastrointestinal (GI) disorders, encompassing oesophageal afflictions, and the multifaceted realm of psychological factors [4–7]. There is a well-established and significant relationship between the delicate balance found in the gastrointestinal system and the complex functioning of the human brain. For instance, it is well-documented that stress and mood exert a palpable influence on the functionality of the GI system, and play a pivotal role in shaping the onset of GI symptoms and disorders[8]. Several observational studies on GERD have suggested that psycho-emotional aspects play a major role in the lives of those who suffer from this illness [9–11]. While the aforementioned evidence does hint at a plausible link between psycho-emotional disorders and GERD, it’s essential to acknowledge the limitations inherent in these observational studies. These constraints include the potential for residual confounding and reverse causality, which can obscure our understanding of causality. Consequently, the precise nature of the causal relationship between psycho-emotional disorders and GERD remains unclear.

In recent times, Mendelian Randomization (MR) studies have garnered increasing scholarly attention. MR presents a uniquely well-suited statistical approach for epidemiological investigations that seeks to identify the causal relationships between exposures and outcomes. The strength of MR lies in its capacity to sidestep the challenges of confounding and reverse causation simultaneously. This is achieved by leveraging genetic variants, which are randomly assigned at conception, typically remain unaffected by environmental risk factors, and precede the onset of the disease [12]. To our current knowledge, there is an absence of MR studies that have examined the causation linking GERD and psycho-emotional disorders. Therefore, through this study, we can determine the causal relationship between gastroesophageal reflux and psychological and emotional disorders. It can be concluded that effective management of high-risk groups and patients’ psychological emotions is conducive to the prevention and treatment of GERD.

## Materials and methods

### Data sources

The genome-wide association study (GWAS) pooled statistics concerning GERD that are employed in this investigation were procured from the FinnGen Consortium [13], which included 13,141 European patients with GERD and 189,695 healthy controls. The datasets about mood swings, highly strung, and anxious feelings were sourced from the European sample cohorts that are included in the Open-GWAS database. These datasets were subject to analysis by the Neale Lab and Ben Elsworth [14]. For our investigation, we accessed and analyzed large-scale information pertaining to mood swings from a large sample of 329,428 European people, including 180,827 controls and 148,601 cases. Additionally, data on highly strung were culled from a substantial cohort of 447,961 European individuals, featuring 78,408 cases and 189,695 controls. Furthermore, data on anxious feelings were gleaned from a robust dataset involving 450,765 European individuals, presenting 255,812 cases and 194,953 controls. Table 1 furnishes more details regarding the summary-level data from the GWAS encompassing the exposure and outcome variables central to our MR study. These datasets were obtained from the IEU Open GWAS project (https://gwas.mrcieu.ac.uk), and each cohort underwent rigorous ethical approval with explicit informed consent obtained from all participants.

**Table 1:** Detailed information on GWAS data.

Traits	N case	N control	Population	Data accession address
GERD	13141	189695	European	https://gwas.mrcieu.ac.uk/
Mood swings	148601	180827		
Highly strung	78408	369553		
Anxious feelings	255812	194953		

Abbreviation: GERD, gastroesophageal reflux disease

### Selection of instrumental variables (IVs)

MR analyses harness instrumental variables (IVs) to scrutinize the causal relationship between an exposure and an outcome. The selection of single nucleotide polymorphisms (SNPs) as exposure IVs necessitates strict adherence to three fundamental assumptions: (1)

**Fig 1:**
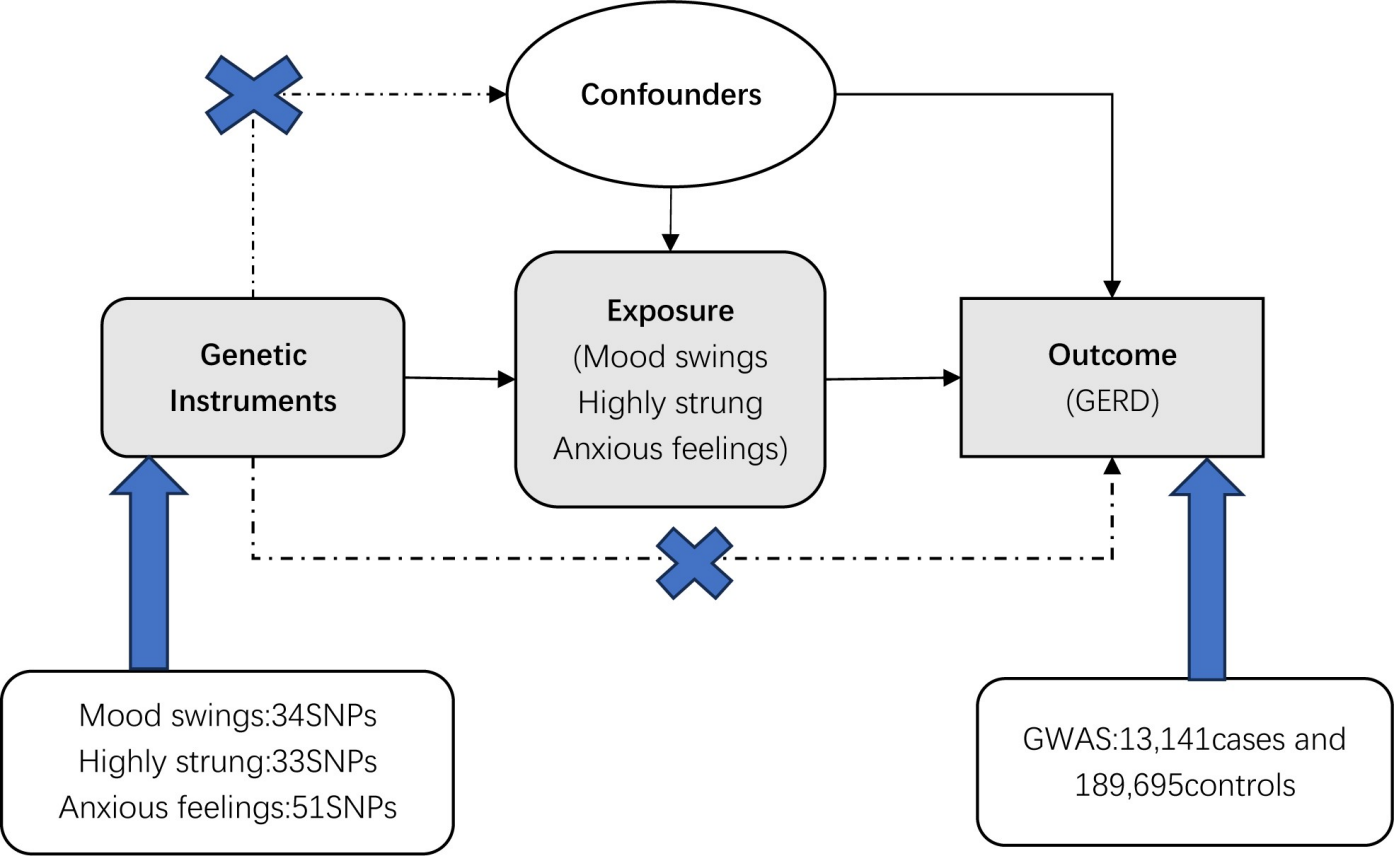
Selection of IVs for MR.

Strength of Association: The chosen SNPs must exhibit robust and unequivocal associations with the exposure. This foundational criterion underscores the pivotal role of SNPs as effective instruments for assessing causality; (2) Absence of Confounding: The selected SNPs must remain uncorrelated with any potential confounding variables that might distort the true association between the exposure and the outcome; and (3) Causality Unidirectionality: The SNPs employed as instruments should exclusively influence the outcome through their impact on the exposure. This criterion establishes the causal pathway and guards against spurious associations [15]. (Fig 1) In sum, these stringent prerequisites underpin the reliability and validity of MR analyses.

Adhering to the foundational principles outlined above, our initial step involved a meticulous screening process. we first screened 40, 41, and 67 SNPs associated with mood swings, and highly strung and anxious feelings, respectively, at the genome-wide significance threshold (P < 5×10−8) for MR analysis. Furthermore, we ensure that all SNPs linked to the exposure variables remained independent, devoid of any discernible linkage di-sequilibrium (LD) within a range of 10,000 kilobases, with a squared correlation coefficient (R2) less than 0.001. This meticulous approach upholds the integrity of our MR framework, guaranteeing the autonomy of each instrumental variable. Additionally, we recognized smoking, alcohol consumption, and body mass index (BMI) as pertinent variables with the potential to confound the exposure-outcome relationship [16–20]. We systematically addressed potential confounding factors by excluding the SNPs associated with these variables(P < 5×10−8). This process was conducted through the online platform (http://www.phenoscanner.medschl.cam.ac.uk/). Furthermore, we exercised prudence by excluding palindromic SNPs, SNPs exhibiting associations with the outcome variable at a significance level of P<0.05, and SNPs that were absent in the GWAS about the outcome variable. This discerning approach yielded a refined set of 34, 33, and 51 IVs closely linked to mood swings, highly strung, and anxious feelings, respectively. We have documented the excluded SNPs, along with the precise rationales for their exclusion, as well as comprehensive characteristics of the SNPs that successfully navigated our selection criteria. This information is cataloged in the Supporting information.

Lastly, we subjected the IVs to a thorough assessment of their strength, gauged by the F-statistic, which yielded values spanning from 29.75 to 50.68. This comprehensive evaluation conclusively established the absence of weak instrumental variables, reaffirming the robustness and reliability of our instrumental variable selection process [21, 22].

### Statistical methods

We used the random effects model with the IVW method as the main statistical analysis [23]. It is an extension of the Wald ratio estimator based on the principles of meta-analysis, which does not take into account the presence or absence of an intercept term in the regression and employs a weighted linear regression that forces the intercept to be zero, which improves the precision and testability of the estimation when the IVs satisfy the three main assumptions [24]. The significance threshold was set at P < 0.05, and causal association results were expressed as odds ratios(OR) and 95% confidence intervals (95% CI). The method weights each ratio according to its standard error and takes into account possible heterogeneity [15]. In addition to the IVW method, the MR-Egger and Weighted Median Method (WME) were added as a complement to assess causality [25, 26]. Because of its stability and accuracy in the absence of directional pleiotropy, the traditional IVW method was used as the primary MR analysis for assessing the relationship between psychoemotional disorders and GERD [27]. The WME can furnish accurate results even when more than half of the instrumental variables face potential invalidation [26]. WME reduces the risk of type I errors, thus ensuring a discerning and precise appraisal of causality even in the presence of this intricate genetic phenomenon [26]. MR-Egger regressions are not affected by the validity of IVs [25]. We then conducted a series of sensitivity analyses. Heterogeneity was first assessed using Cochran’s Q-test. Then, the MR-Egger intercept and MRPRESSO global tests were used to detect horizontal polytropy. Finally, leave-one-out sensitivity analyses were performed to assess the robustness of the results. All MR analyses were performed in R (version 4.2.3) using TwoSampleMR (version 0.5.7) and MRPRESSO (version 1.0).

## Results

**Fig 2:**
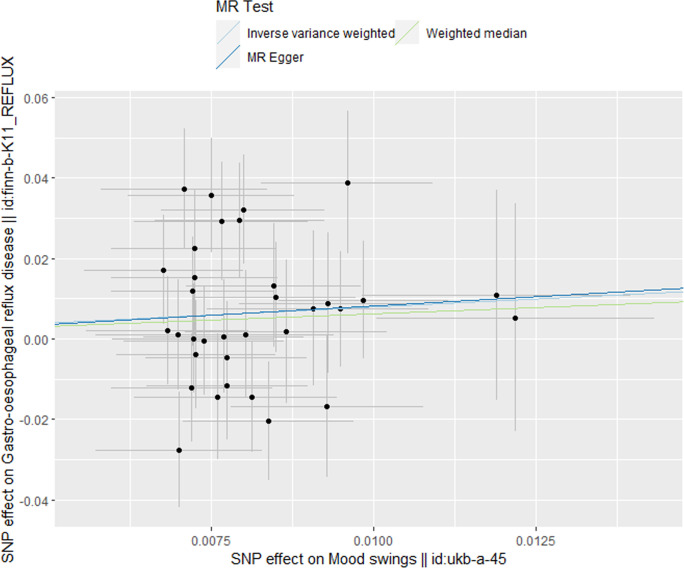
Scatterplot of MR analysis to assess the causal relationship between mood swings and GERD.

**Fig 3:**
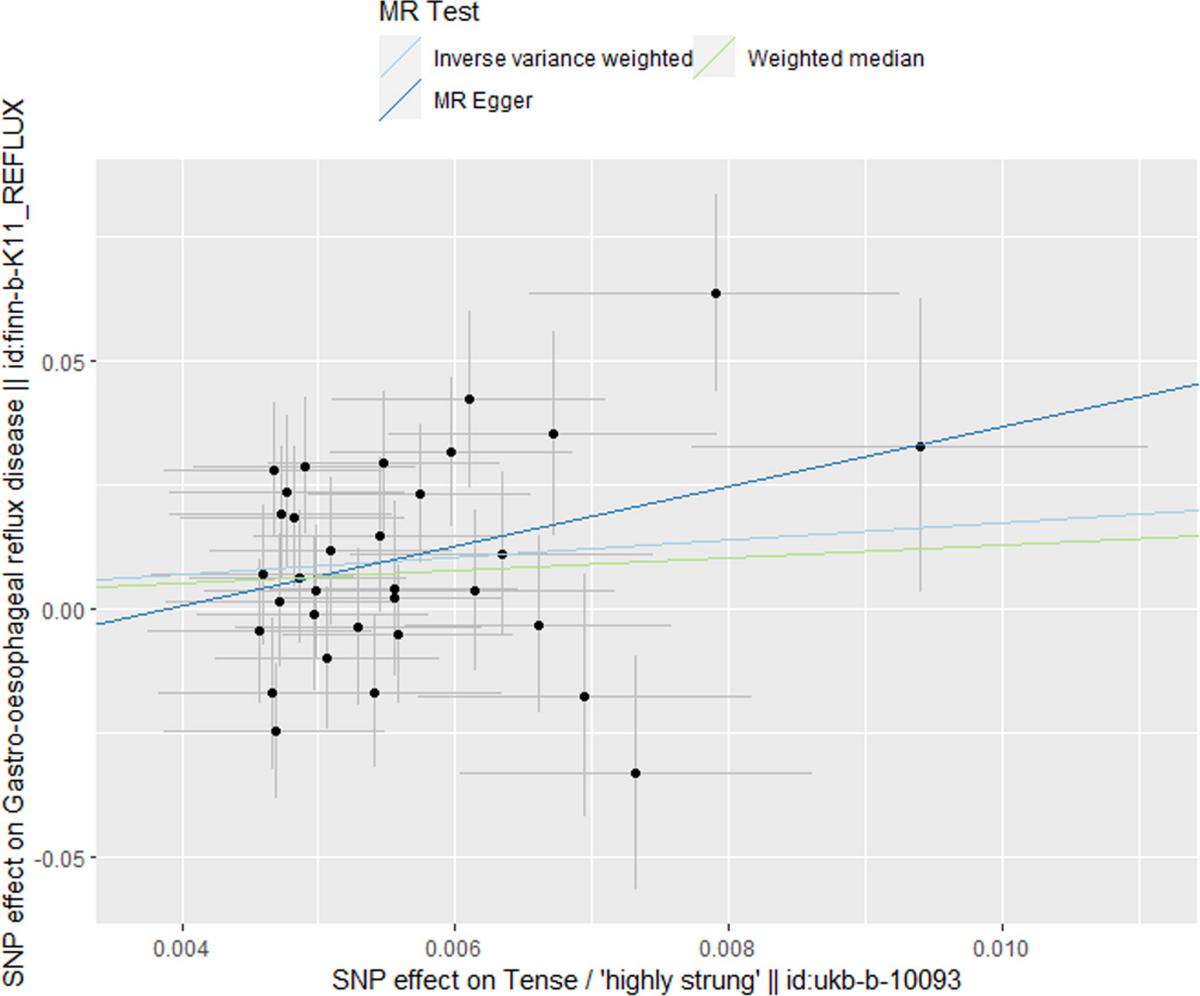
Scatterplot of MR analysis to assess the causal relationship between highly strung and GERD.

**Fig 4:**
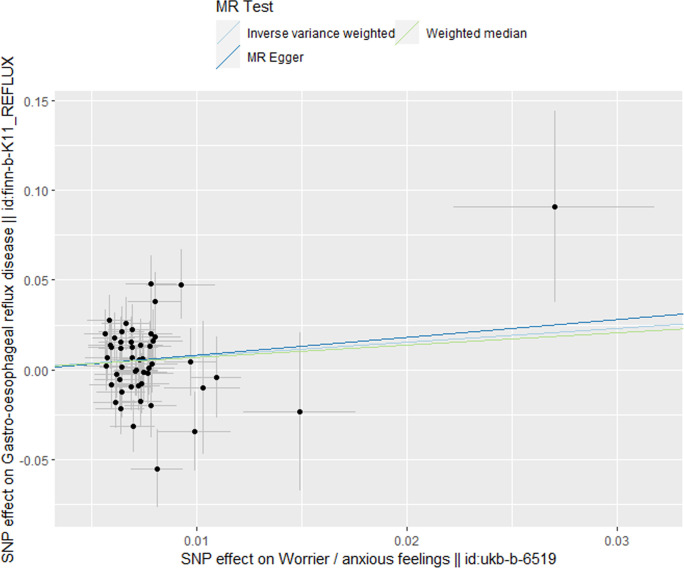
Scatterplot of MR analysis to assess the causal relationship between anxious feelings and GERD.

**Fig 5:**
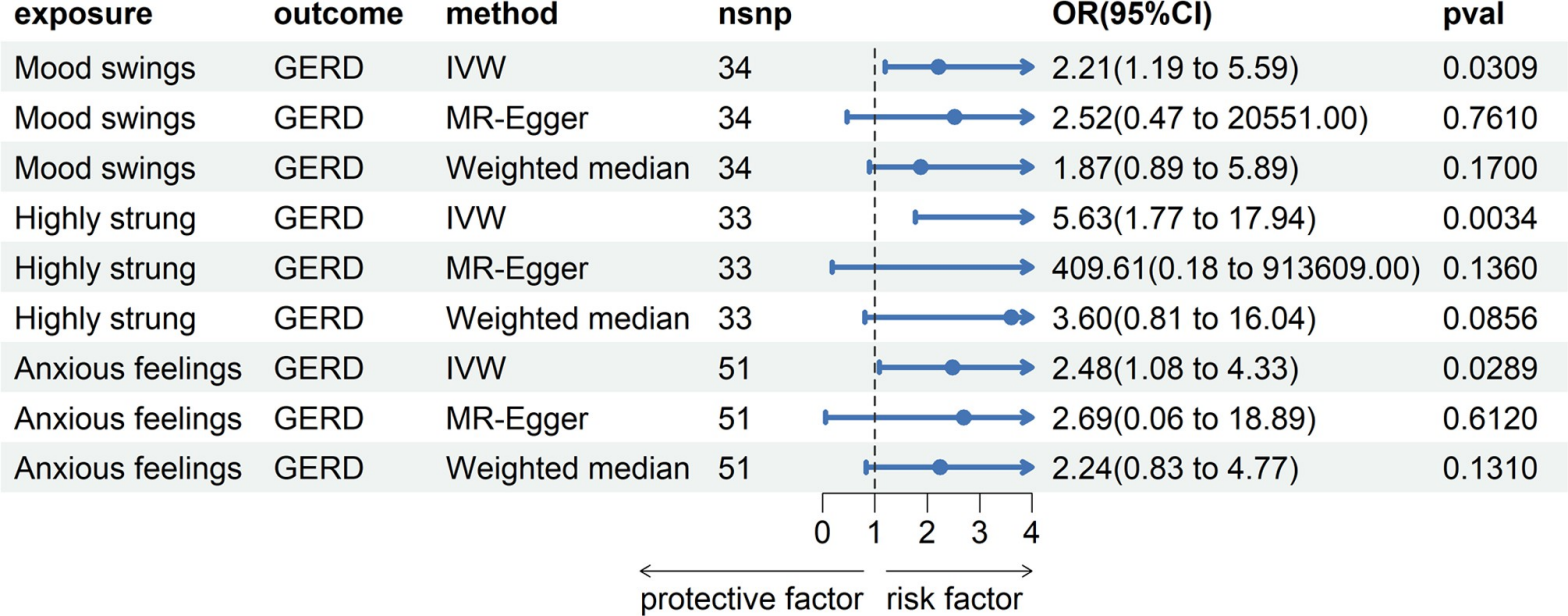
Forestplot of associations between exposures and risk of GERD in MR analysis.

MR analysis conducted in our study revealed a causal relationship between psychoemotional disturbances and GERD. Specifically, Mood swings, highly strung and anxious feelings adversely affected GERD risk (mood swings: OR 2.21, 95% CI 1.19–5.59, p = 3.09 × 10–2; highly strung: OR 5.63, 95% CI 1.77–17.94, p = 3.42 × 10–3; anxious feelings: OR 2.48, 95% CI 1.08–4.33, p = 2.89 × 10–2)(Figs 2–5). It is noteworthy that although the results derived from our weighted median approach did not attain statistical significance (P>0.05), the direction of the estimated effect consistently leaned towards the OR greater than 1. Detailed data are presented in Table 2. Additionally, the substantial number of SNPs used in our MR analysis serves as a testament to the robustness of our findings, bolstering their credibility.

**Table 2:** The causal relationship between mood swings, highly strung, anxious feelings and GERD in Mendelian randomization.

Exposure	Outcome	n SNP	Method	OR (95% CI)	P-value
Mood swings	GERD	34	IVW	2.21 (1.19–5.59)	3.09 × 10−2
			MR-Egger	2.52 (0.47–20551)	7.61 × 10−1
			Weighted median	1.87 (0.89–5.89)	1.70 × 10−1
Highly strung		33	IVW	5.63 (1.77–17.94)	3.43 × 10−3
			MR-Egger	409.61 (0.18–913609)	1.36 × 10−1
			Weighted median	3.60 (0.81–16.04)	8.56 × 10−2
Anxious feelings		51	IVW	2.48 (1.08–4.33)	2.89 × 10−2
			MR-Egger	2.69 (0.06–18.89)	6.12 × 10−1
			Weighted median	2.24 (0.83–4.77)	1.31 × 10−1

Abbreviations: Cl, confidence interval; IVW, inverse variance weighted; OR, odds ratio

To further fortify the validity of our MR results, we conducted a battery of sensitivity analyses. Cochran’s Q test yielded no evidence of heterogeneity (P>0.05) among the instrumental variables in the MR analyses for both mood swings and highly strung (Table 3). While a mild degree of heterogeneity surfaced in the case of anxious feelings (P<0.05), our utilization of the IVW random effects model ensured the resilience of our results. Subsequent examination using MR-PRESSO did detect outliers in the analyses of anxiety and GERD. However, the Distortion Test unveiled no significant outliers, and the results from the MR-Egger Intercept Test indicated that the analysis remained impervious to the potential influence of horizontal pleiotropy (P>0.05). Consequently, we maintain confidence in positing that feelings of anxiety bear a positive causal effect on GERD. Furthermore, the MR-Egger intercept test and the MRPRESSO global test conducted in the analyses about mood swings and highly strung substantiated the absence of horizontal pleiotropy (P>0.05), further enhancing the robustness of our conclusions (Table 4).

**Table 3:** Heterogeneity of mood swings, highly strung, anxious feelings and GERD in MR analysis.

Exposure	Outcome	Method	Cochran’s Q test		
			Q	Q_df	Q_pval
Mood swings	GERD	IVW	43.66	33	0.102
		MR-Egger	43.65	32	0.082
Highly strung		IVW	45.51	32	0.057
		MR-Egger	43.79	31	0.063
Anxious feelings		IVW	68.95	50	0.039
		MR-Egger	68.93	49	0.032

**Table 4:** Mood swings, highly strung, anxious feelings, and GERD in MR analysis of horizontal pleiotropy.

Exposure	Outcome	MR-Egger intercept test			MR-PRESSO global test	
		Intercept	SE	P-value	RSS obs	P-value
Mood swings	GERD	-0.001	0.024	0.965	46.29	0.116
Highly strung		-0.023	0.021	0.279	48.43	0.062
Anxious feelings		-0.002	0.138	0.911	71.69	0.036

**Fig 6:**
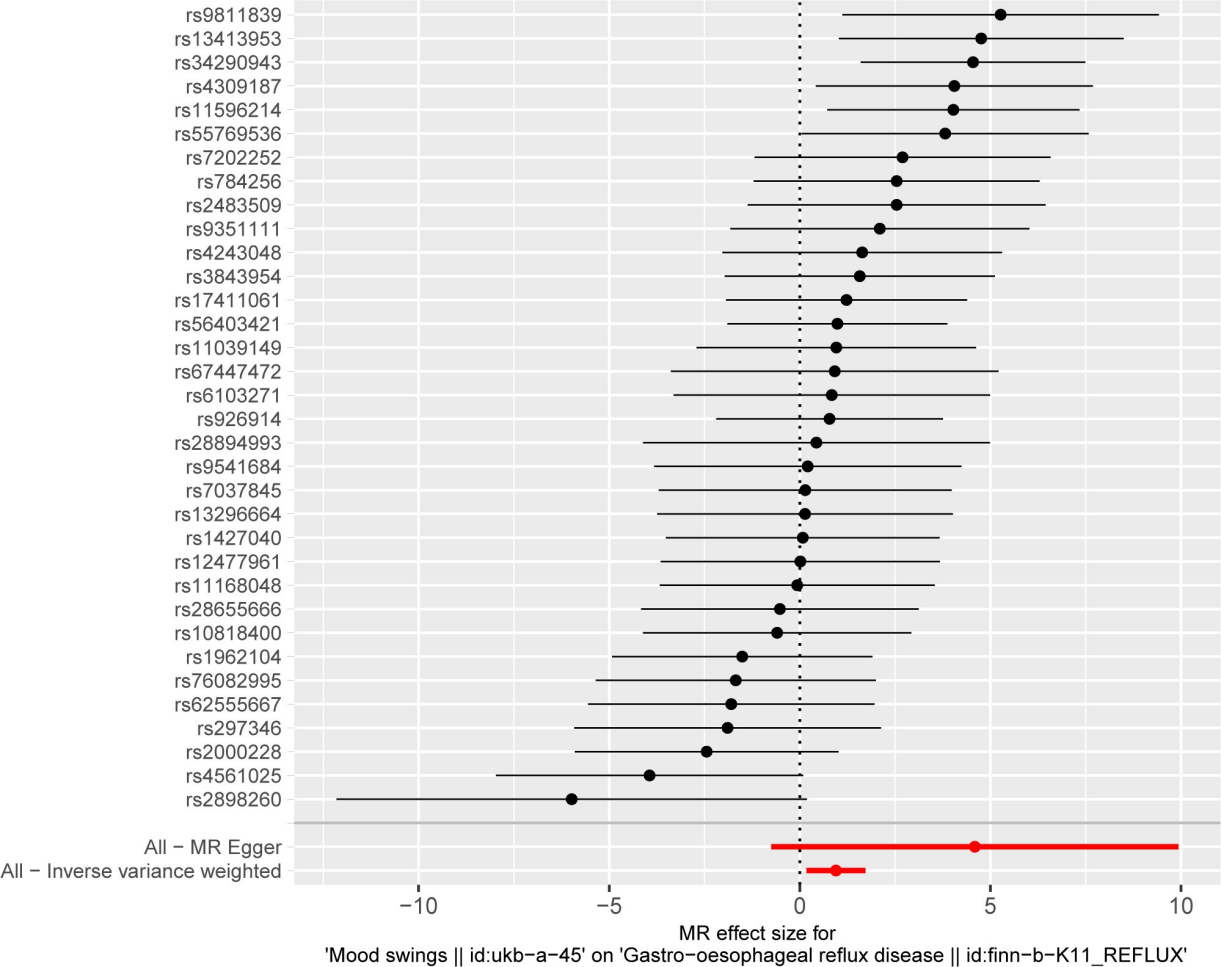
The leave-one-out method of the causal relationship between mood swings and GERD.

**Fig 7:**
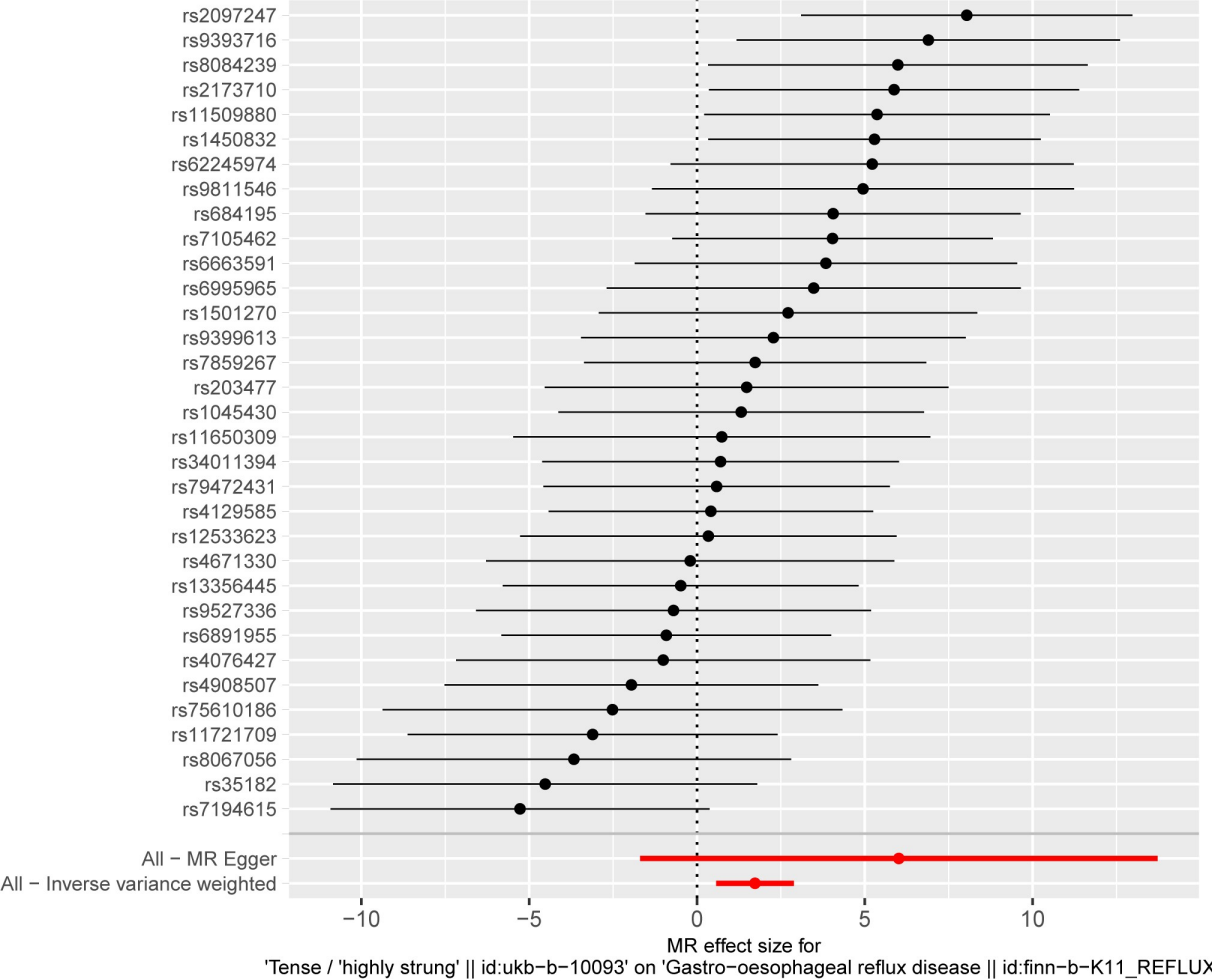
The leave-one-out method of the causal relationship between highly strung and GERD.

**Fig 8:**
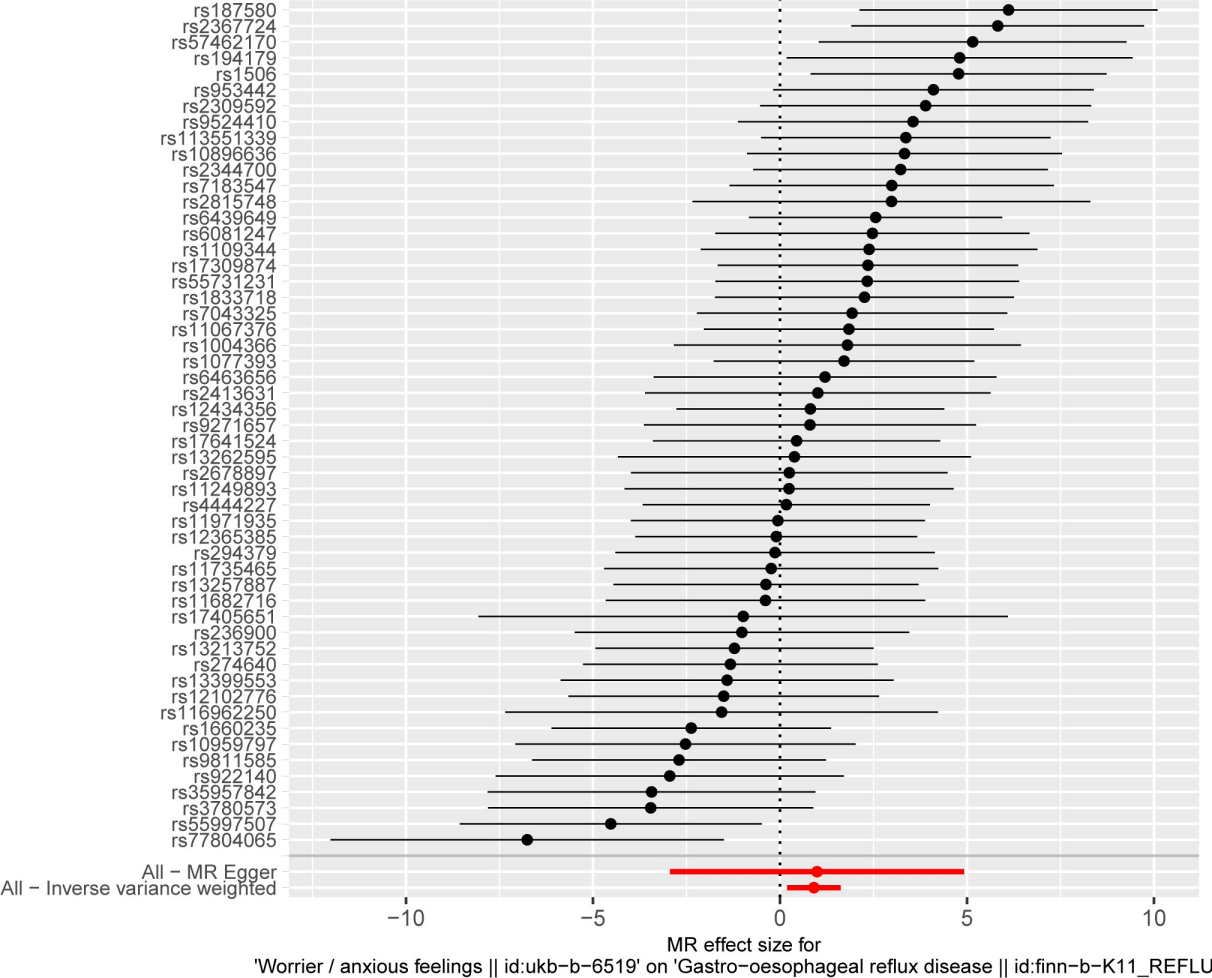
The leave-one-out method of the causal relationship between anxious feelings and GERD.

Finally, leave-one-out sensitivity analyses were executed, confirming the steadfastness of our MR results. Even after the removal of individual SNPs from the analysis, no major SNP emerged as a significant determinant significantly altering the results (Figs 6–8). This corroborates the stability and integrity of our findings, reinforcing the notion of a causal link between psychoemotional disturbances and GERD.

## Discussion

In the current investigation, we harnessed the power of MR analysis to elucidate and substantiate a causal nexus between psycho-emotional disorders and GERD. Our study proves that mood swings, highly strung and anxious feelings are positively associated with GERD. Besides, these findings were robustly upheld when subjected to sensitivity analyses, thereby underlining the reliability and steadfastness of our conclusions.

The results of our study are in line with previous research efforts in this field and have resonance in the wider scientific community. An example is the work of Kessing et al., whose meticulous examination encompassed a cohort of 225 patients exhibiting symptoms indicative of GERD. Their findings unveiled a positive correlation between elevated levels of anxiety and the heightened severity of GERD symptoms. Moreover, this interplay between psycho-emotional states and GERD symptomatology was shown to impede the overall quality of life [28]. A cross-sectional study including 4790 patients with psychosocial disorders and 728,749 with GERD found that a diagnosis of a psychological disorder significantly increased the risk of GERD (OR, 3.16, 95% CI, 2.71–3.68) [29]. Jansson et al. observed and quantified the impact of psychological factors on the occurrence of reflux symptoms. Their discerning analysis revealed that anxiety increased the risk of reflux symptoms ([OR]3.2; 95%[CI], 2.7–3.8; P < 0.0001), whereas depression led to a 1.7-fold increase in risk ([OR]1.7; 95%[CI],1.4–2.1; P < 0.0001) [30]. While these observational studies do not elucidate causality, they nonetheless furnish compelling evidence supporting a discernible connection between GERD and psychological disorders.

The following are the mechanisms by which psycho-emotional disorders increase the risk of developing GERD. First and foremost, evidence has emerged from research conducted on psychologically stressed rats, demonstrating a link between psychological stress and the integrity of oesophageal epithelial tight junctions [31]. Secondly, it is worth noting that mental states, exemplified by anxiety, have the potential to exert deleterious effects on oesophageal motor function. Specifically, these psychological states can lead to a reduction in the pressure exerted on the lower oesophageal sphincter, consequently giving rise to oesophageal dysmotility [32]. Thirdly, psychological disorders can affect oesophageal sensitivity through peripheral and central mechanisms; i.e., peripheral sensitization and central sensitization. Central sensitization plays a crucial role in oesophageal hypersensitivity. That is, mechanical and chemical stimuli are converted into action potentials by nociceptors on the oesophageal nerves, which are then transmitted to the central nervous system via the spinal or vagus nerves, causing excitatory synaptic responses, which in turn enhance the patient’s sensitivity to physiological stimuli [33]. As a result of the combination of these factors, the increased psychosocial barriers put the risk of GERD at a correspondingly higher level. This holistic understanding of the interplay between psychosocial factors and GERD not only deepens our comprehension of disease etiology but also underscores the importance of addressing psychological well-being in the context of gastroesophageal health.

Our investigation presents a constellation of notable strengths. First, the most prominent advantage of MR Research is to reduce reverse causation and control for confounding factors. Our MR analysis design was based on the three cardinal assumptions that control the selection of IVs and are the sine qua non of MR research. It was carefully constructed while adhering to the fundamental principles of MR methodology. Besides, MR studies bear the imprimatur of heightened validity compared to randomized controlled trials. Furthermore, because its data comes from the Open-GWAS database, there is no ethical restriction. Moreover, the demographic stratification bias was lessened because all of the included subjects were of European heritage. Lastly, to the best of our knowledge, this is the first MR Survey to evaluate the causal connection between GERD and psycho-emotional illnesses, enhancing and improving the findings of earlier pertinent research.

Nevertheless, it is imperative to acknowledge the inherent limitations of this study. Firstly, it is crucial to recognize that the primary GWAS summary data used in our analysis originated from a European population. This caveat underscores the necessity for caution when extrapolating our findings to individuals of diverse ethnic backgrounds, as genetic nuances may engender distinct outcomes across populations. Secondly, we were unable to do subgroup analyses, such as breaking down the GERD population by subtype, sex, or ethnicity, because of the constraints of the GWAS aggregated data. Thirdly, since different statistical techniques cannot rule out horizontal pleiotropy, it should also be a primary concern of MR. In response, we have undertaken a series of sensitivity analyses to bolster the veracity and robustness of our findings, thereby buttressing their credibility. This MR investigation illuminates that heightened levels of GERD have exhibited a positive correlation with mood swings, highly strung, and anxious feelings.

This suggests that we should pay more attention to the impact of mental health factors on GERD. People with highly sensitive personalities who are prone to stress and anxiety ought to be proactive in scheduling screening exams and taking preventative actions to avoid developing GERD. For instance, give up drinking and smoking, and adopt healthy eating practices including chewing food carefully and avoiding eating two to three hours before bed. People who are obese or pregnant are at a higher risk of developing gastric reflux disease (GERD). It is important to monitor these individuals’ psychological mood swings and anxiety levels. Prompt psychological intervention can help lower the risk of GERD. In addition, it is important to properly treat the psycho-emotional health of GERD patients. All things considered, this research offers fresh perspectives on GERD therapy and prevention.

## Supporting information

S1 Table Description of exposure and ending variables. (XLSX)

S2 Table Description of confounding factors. (XLSX)

S1 File Funnel plot of Mendelian analysis of mood swings and GERD. (PDF)

S2 File Funnel plot of Mendelian analysis of highly strung and GERD. (PDF)

S3 File Funnel plot of Mendelian analysis of anxious feelings and GERD. (PDF)
