# Supplementary figures and images for: The causal role of multiple psycho-emotional disorders in gastroesophageal reflux disease: A two-sample Mendelian randomized study
Source: PLoS One. 2024 May 6;19(5):e0302469. doi: 10.1371/journal.pone.0302469 (PMC11073702; doi:10.1371/journal.pone.0302469)

# MR Method

- Inverse variance weighted
- MR Egger

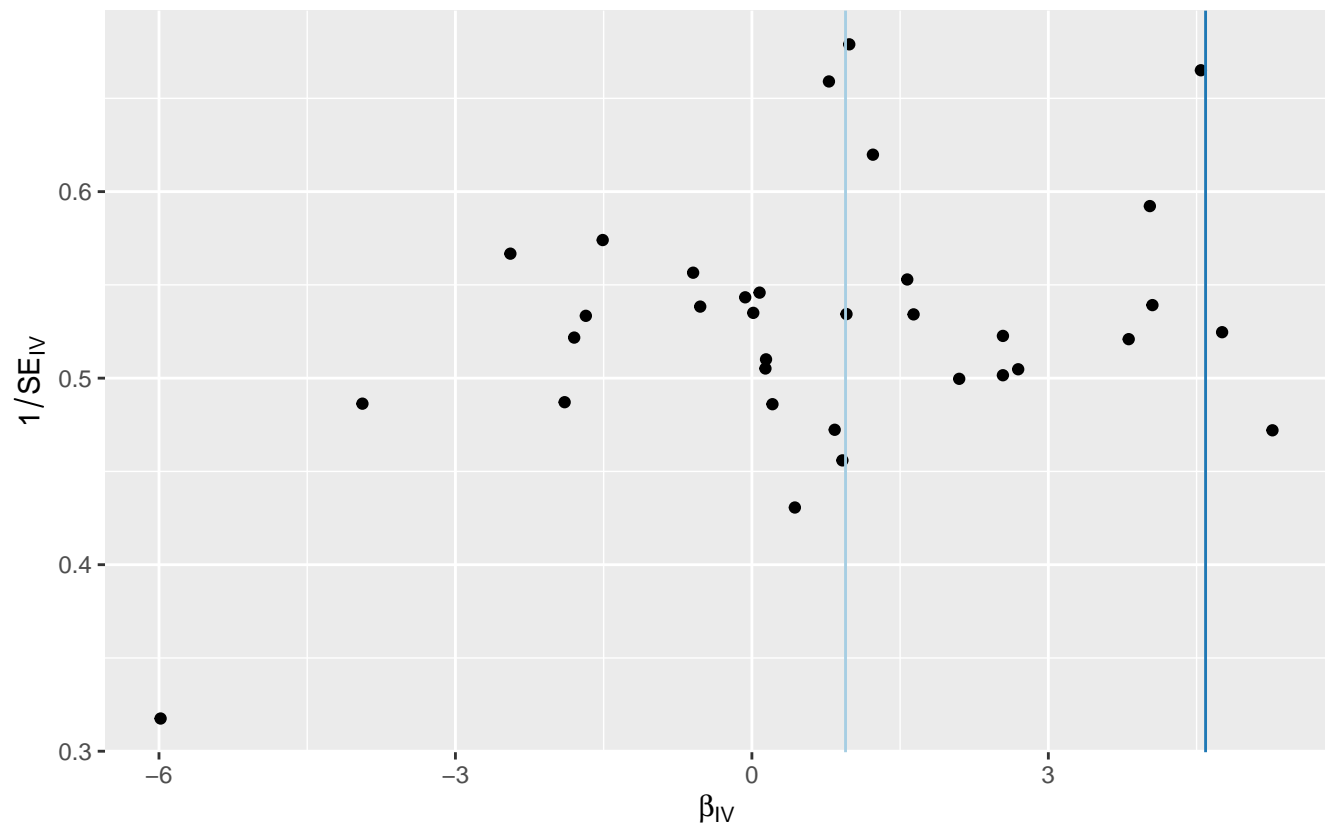

Supplement: S1 File — (PDF) [file pone.0302469.s003.pdf]

# MR Method

- Inverse variance weighted
- MR Egger

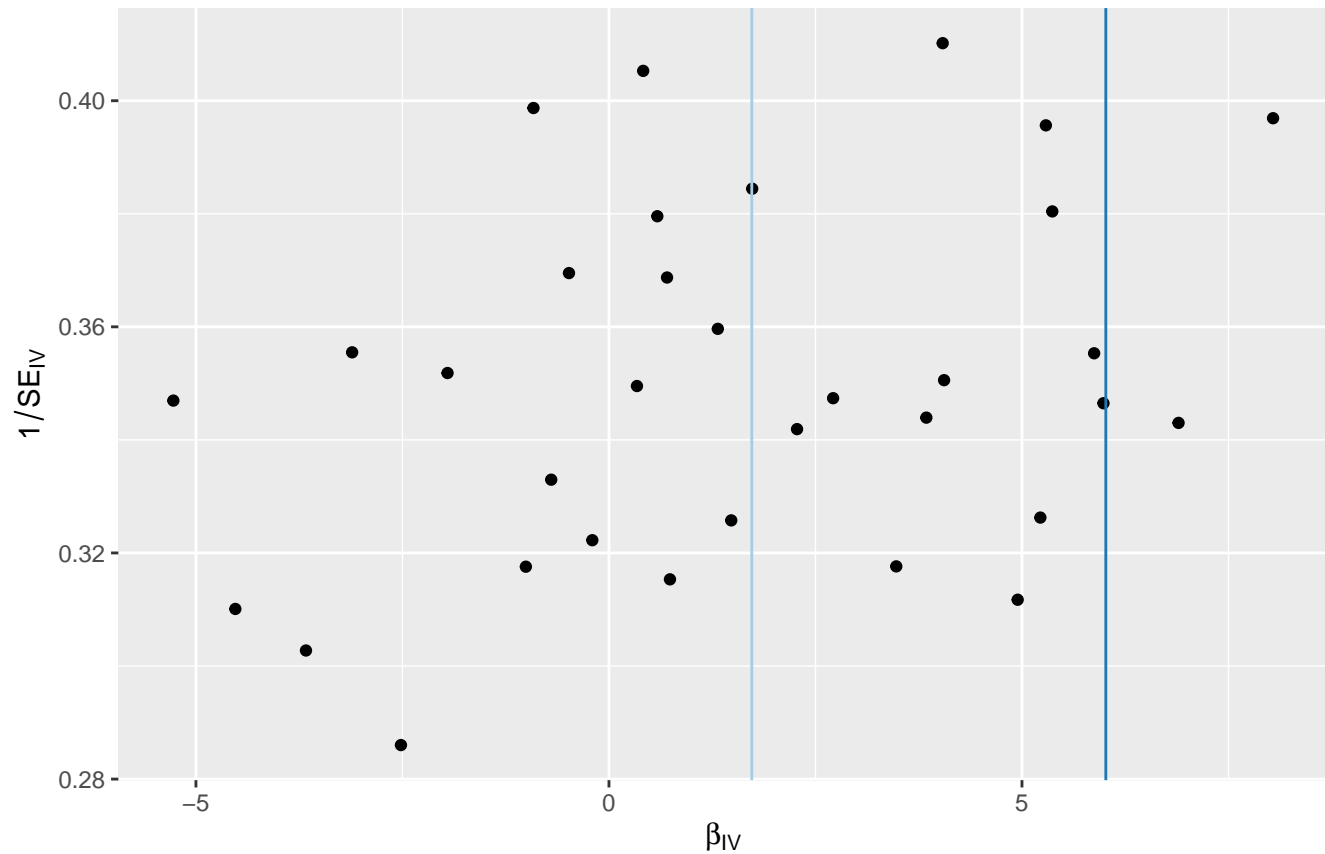

Supplement: S2 File — (PDF) [file pone.0302469.s004.pdf]

# MR Method

- Inverse variance weighted
- MR Egger

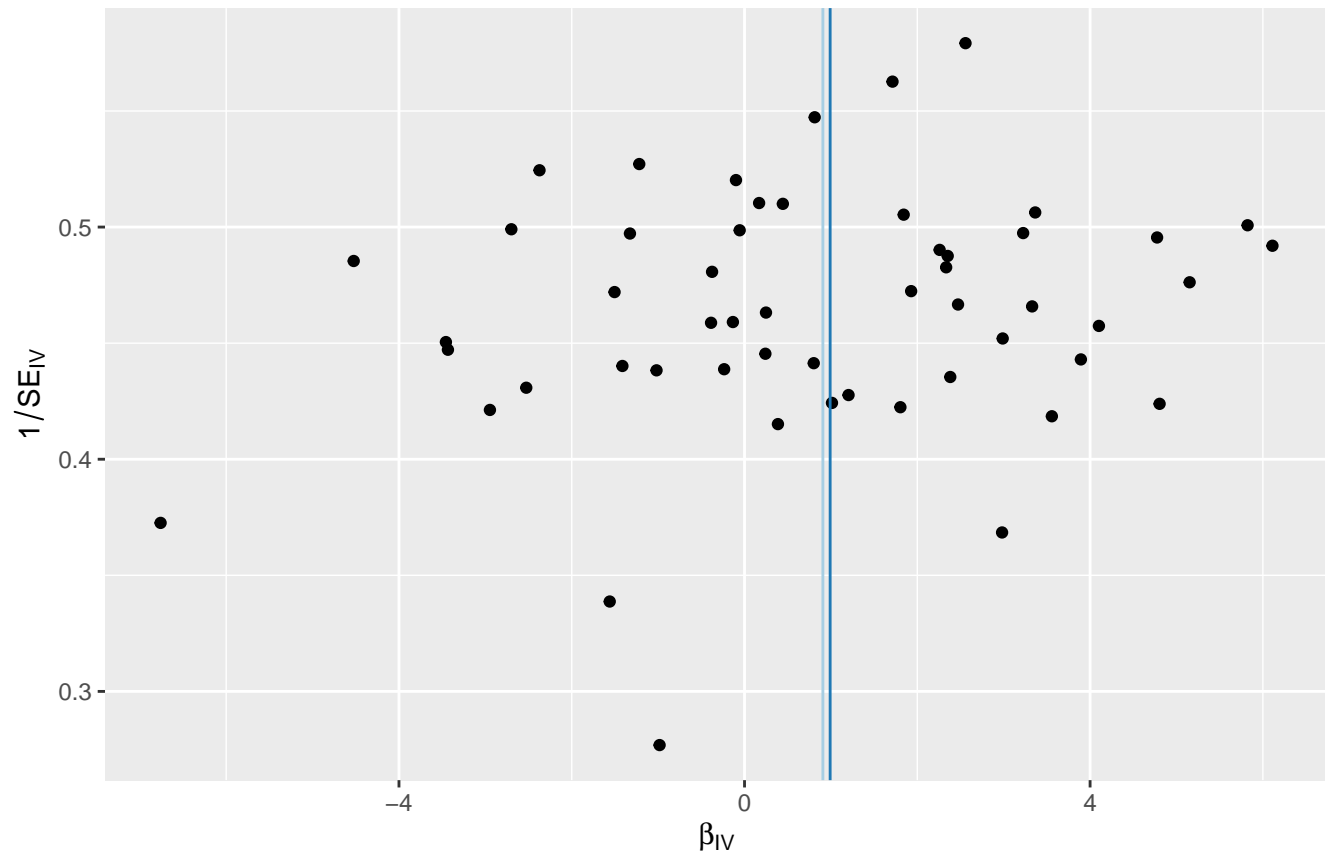

Supplement: S3 File — (PDF) [file pone.0302469.s005.pdf]
